# Dasatinib-Induced Bilateral Chylothorax: A Case Report

**DOI:** 10.7759/cureus.68479

**Published:** 2024-09-02

**Authors:** Caitlin E McGonegal, Sujatha Baskar

**Affiliations:** 1 Osteopathic Medicine, Lake Erie College of Osteopathic Medicine, Bradenton, USA; 2 Internal Medicine, AdventHealth Waterman, Tavares, USA

**Keywords:** philadelphia chromosome positive, bilateral pleural effusion, acute dyspnea, bcr-abl positive, exudative pleural effusion, chronic myeloid leukemia (cml), #dasatinib, bilateral chylothorax

## Abstract

Dasatinib, a BCR-ABL tyrosine kinase inhibitor, is used in the management of Philadelphia-positive chronic myeloid leukemia (CML). Several adverse complications of this targeted immunotherapy have been reported. This case report focuses on a 79-year-old female who presented with acute dyspnea and generalized chest pressure while undergoing management with this specific tyrosine kinase inhibitor. Bilateral chylothorax was diagnosed with the aid of imaging, laboratory studies, and diagnostic thoracentesis.

No other risk factors, including trauma, lung malignancies, or congenital anomalies, were detected in this patient. Since no other etiologies for the development of chylothorax were identified, it was concluded that dasatinib therapy was the inciting factor. Dasatinib was discontinued and bosutinib was initiated. A low-fat diet was prescribed, which the patient was amenable to. Six months later, the patient remained stable with no recurrence of chylothorax. The mechanism of dasatinib-induced chylothorax is currently under investigation. The purpose of this report is to raise awareness about dasatinib-induced chylothorax, aid in identifying predisposing risk factors, and enhance understanding of the proper management of this rare complication.

## Introduction

Dasatinib, sold under the brand name Sprycel, is a BCR-ABL tyrosine kinase inhibitor often utilized in the treatment of chronic myeloid leukemia (CML) [1]. CML is a malignancy of mature myeloid stem cells, particularly granulocytes. It is most commonly diagnosed in middle-aged individuals and the elderly, with an average age at diagnosis of 64 years, and is typically idiopathic in nature [1]. Additionally, exposure to radiation and aging are a few predisposing factors to the development of this hematologic malignancy [1]. A genetic abnormality leading to reciprocal translocation between chromosomes 9 and 22 results in the formation of BCR-ABL oncoprotein. This genetic translocation leads to persistent activation of tyrosine kinase, ultimately promoting rapid, uncontrolled proliferation of myeloid stem cells [2].

Approximately 90% of patients with CML test positive for the Philadelphia chromosome, reflecting the need for early intervention [3]. The development of tyrosine kinase inhibitors aided in the interference and termination of this mutated signal transduction mechanism. Tyrosine kinase inhibitors are proven to induce high rates of remission in patients positive for the Philadelphia chromosome in CML, ultimately reducing mortality from this malignancy [1]. Dasatinib, a tyrosine kinase inhibitor taken orally, plays a vital role in inhibiting additional active and inactive forms of ABL kinase domains, which makes this chemotherapeutic agent useful for imatinib-resistant CML [4]. The commonly reported adverse effects of dasatinib therapy include hepatotoxicity, anemia, neutropenia, thrombocytopenia, and electrolyte abnormalities [5]. Chylothorax is another rare, yet serious, adverse effect of dasatinib therapy in CML.

Only 14 cases of dasatinib-induced chylothorax have been reported in the literature [6]. Generally, the presentation involves an exudative pleural effusion; however, pleural fluid analysis will demonstrate significant triglyceride accumulation. The underlying mechanism behind dasatinib-induced chylothorax formation is poorly understood and is a particular topic of interest. One potential mechanism involves the ability of dasatinib to induce reactive oxygen species, leading to increased endothelial cell permeability while compromising lymphatic structures [7]. We report a case of a 79-year-old female on dasatinib therapy for CML who presented with a new-onset bilateral chylothorax.

## Case presentation

A 79-year-old Caucasian female presented to the emergency department with a four-day history of acute-onset shortness of breath and dull, non-radiating, generalized chest pressure. The patient’s past medical history was significant for CML, diagnosed in 2023 and under current management with dasatinib therapy, breast cancer status post-lumpectomy and radiation therapy, asthma, and chronic obstructive pulmonary disease. Physical examination on presentation was significant for decreased breath sounds in bilateral lower lobes alongside sinus tachycardia. The remainder of the examination was unremarkable. 

**Figure 1:**
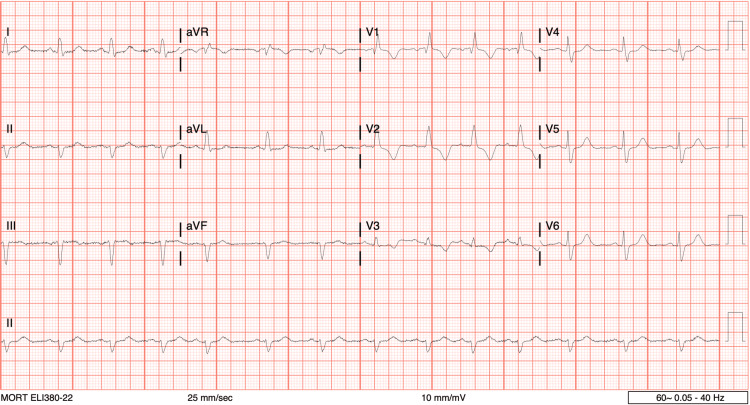
Electrocardiogram at initial presentation The image shows sinus rhythm with right bundle branch block and left axis deviation. No QT prolongation; no STEMI STEMI: ST-segment elevation myocardial infarction

As seen in Figure 1, acute coronary syndrome at presentation was ruled out with an electrocardiogram; meanwhile, sinus rhythm with right bundle branch block and left axis deviation was noted. Table 1 provides the details of the initial laboratory evaluation at admission. The patient's initial troponin was elevated at 15 ng/mL. The remaining repeat troponins remained stable. On presentation, the patient’s white blood cell count was 4.74 k/mcL, but hemoglobin levels were within normal limits at 12.6 g/dL (Table 1). An elevated serum lactate dehydrogenase of 241 U/L was recorded. A lipid panel was also ordered, and results demonstrated a total cholesterol of 164 mg/dL, low-density lipoprotein of 92.0 mg/dL, high-density lipoprotein of 49.00 mg/dL, and triglycerides of 139 mg/dL.

**Table 1:** Labs performed at the initial presentation

Lab findings		
Complete blood count with differentials		
Variable	Patient value	Reference range
White blood cells (WBC/µL)	4.74	3.30-10.60 x10^3^
Red blood cells (RBC/µL)	4.35	3.42-5.40 x10^6^
Hemoglobin (g/dL)	12.6	11.8-16.0
Hematocrit (%)	39.2	35.0-47.0
Mean corpuscular volume (fL)	90.1	80.0-99.0
Mean corpuscular hemoglobin (pg)	29	27.0-33.0
Mean corpuscular hemoglobin concentration (g/dL)	32.1	33.0-36.0
Red blood cell distribution width (%)	14.8	10.3-13.8
Platelet count (platelets/µL)	380	148-426 x10^3^
Mean platelet volume (fL)	9	7.0-10.5
Nucleated red blood cells (%)	0	0
Neutrophils (%)	37.5	50.0-70.0
Lymphocytes (%)	47.3	21.0-45.0
Monocytes (%)	8.2	1.0-15.0
Eosinophils (%)	5.7	0.0-5.0
Basophils (%)	1.1	0.0-3.1
Immature granulocyte (%)	0.2	
Neutrophils absolute (cells/µL)	1.78	1.30-7.20 x10^3^
Lymphocytes absolute (cells/µL)	2.24	1.00-4.90 x10^3^
Monocytes absolute (cells/µL)	0.39	0.30-1.00 x10^3^
Eosinophils absolute (cells/µL)	0.27	0.00-0.40 x10^3^
Basophils absolute (cells/µL)	0.05	0.00-0.30 x10^3^
Immature granulocytes absolute (cells/µL)	0.01	0.00 x10^3^
Comprehensive metabolic panel		
Variable	Patient value	Reference range
Sodium (mmol/L)	141	136-145
Potassium (mmol/L)	4	3.5-5.1
Chloride (mmol/L)	105	101-111
Carbon dioxide (mmol/L)	24	22.0-30.0
Anion gap (mmol/L)	12	4.0-12.0
Blood urea nitrogen (mg/dL)	9	6.0-20.0
Creatinine (mg/dL)	0.6	0.40-1.40
Blood urea nitrogen/creatinine ratio	15	-
Estimated glomerular filtration rate (mL/min/1.73 m^2^)	91.4	75
Glucose (mg/dL)	100	74-100
Calcium (mg/dL)	9.3	8.0-10.2
Magnesium (mg/dL)	2.1	1.70-2.60
Albumin (g/dL)	4.4	3.50-5.20
Globulin (g/dL)	2.9	-
Albumin/globulin ratio	1.5	-
Total protein (g/dL)	7.3	6.6-8.7
Aspartate aminotransferase (U/L)	26	15-41
Alanine aminotransferase (U/L)	11	4-36
Bilirubin, total (mg/dL)	0.3	0.20-1.20
Alkaline phosphatase (U/L)	64	38-126
Cardiac profile		
Variable	Patient value	Reference range
Troponin 1 (ng/L)	15	<14
Troponin 2 (ng/L)	15	<14
Troponin 3 (ng/L)	15	<14
N-terminal Pro-BNP (pg/mL)	158.1	0.0-1800.0
Lipid panel		
Variable	Patient value	Reference range
Cholesterol, total (mg/dL)	164	<200
High-density lipoprotein cholesterol (mg/dL)	49	>41.00
Very low-density lipoprotein, calculated (mg/dL)	28	2-30
Low-density lipoprotein cholesterol, direct (mg/dL)	92	<100.00
Triglycerides (mg/dL)	139	35-160
Other chemistries		
Variable	Patient value	Reference range
Lactate dehydrogenase (U/L)	241	135-225
Osmolality, calculated (mOsm/kg)	270	-
Coagulation panel		
Variable	Patient value	Reference range
Prothrombin time (s)	13.5	11.5-14.1
International normalized ratio	1.03	0.87-1.13

**Figure 2:**
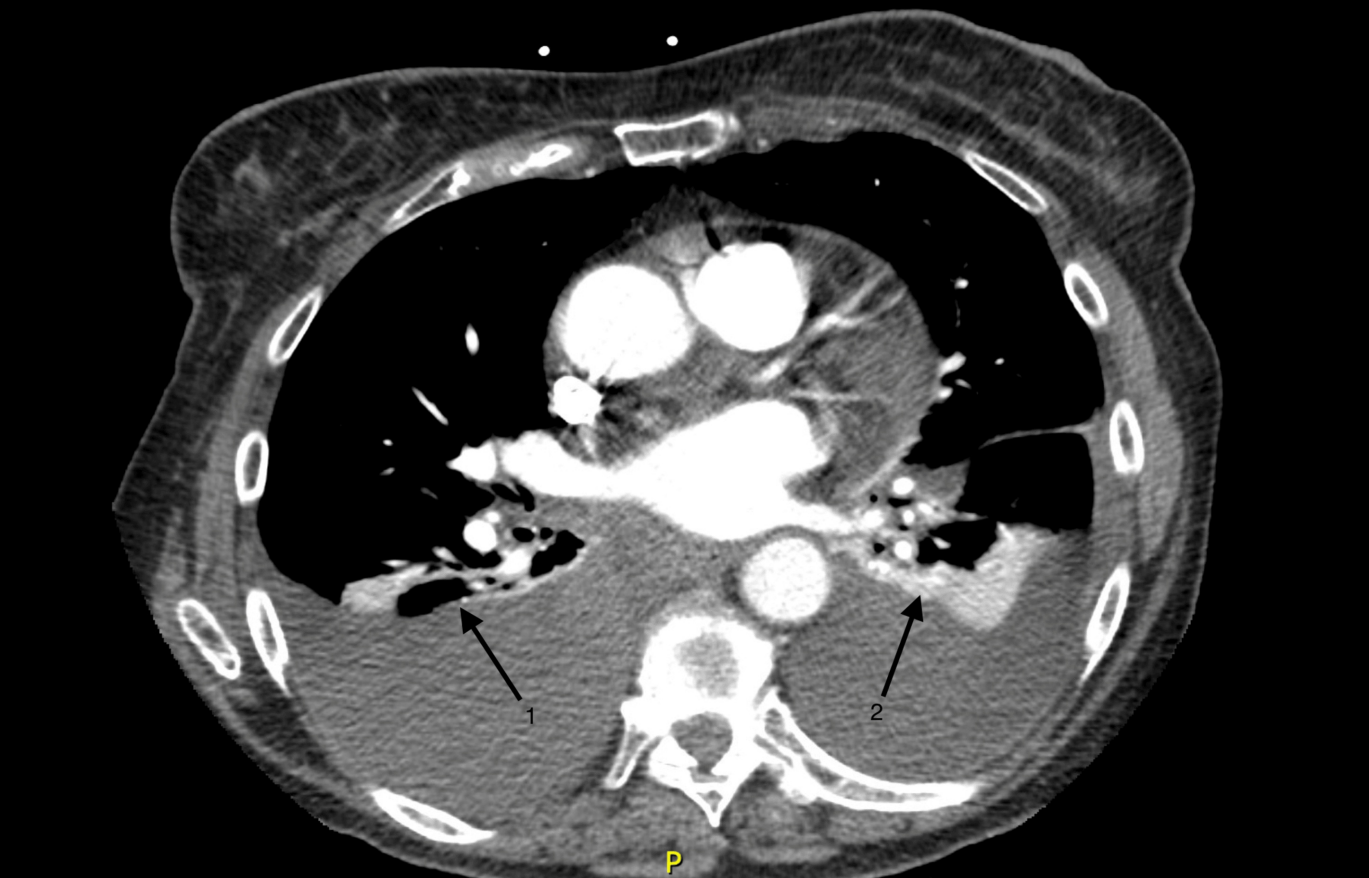
CT chest angiogram Arrows 1 and 2 show bilateral pleural effusions. No pulmonary embolism was visualized CT: computed tomography

**Figure 3:**
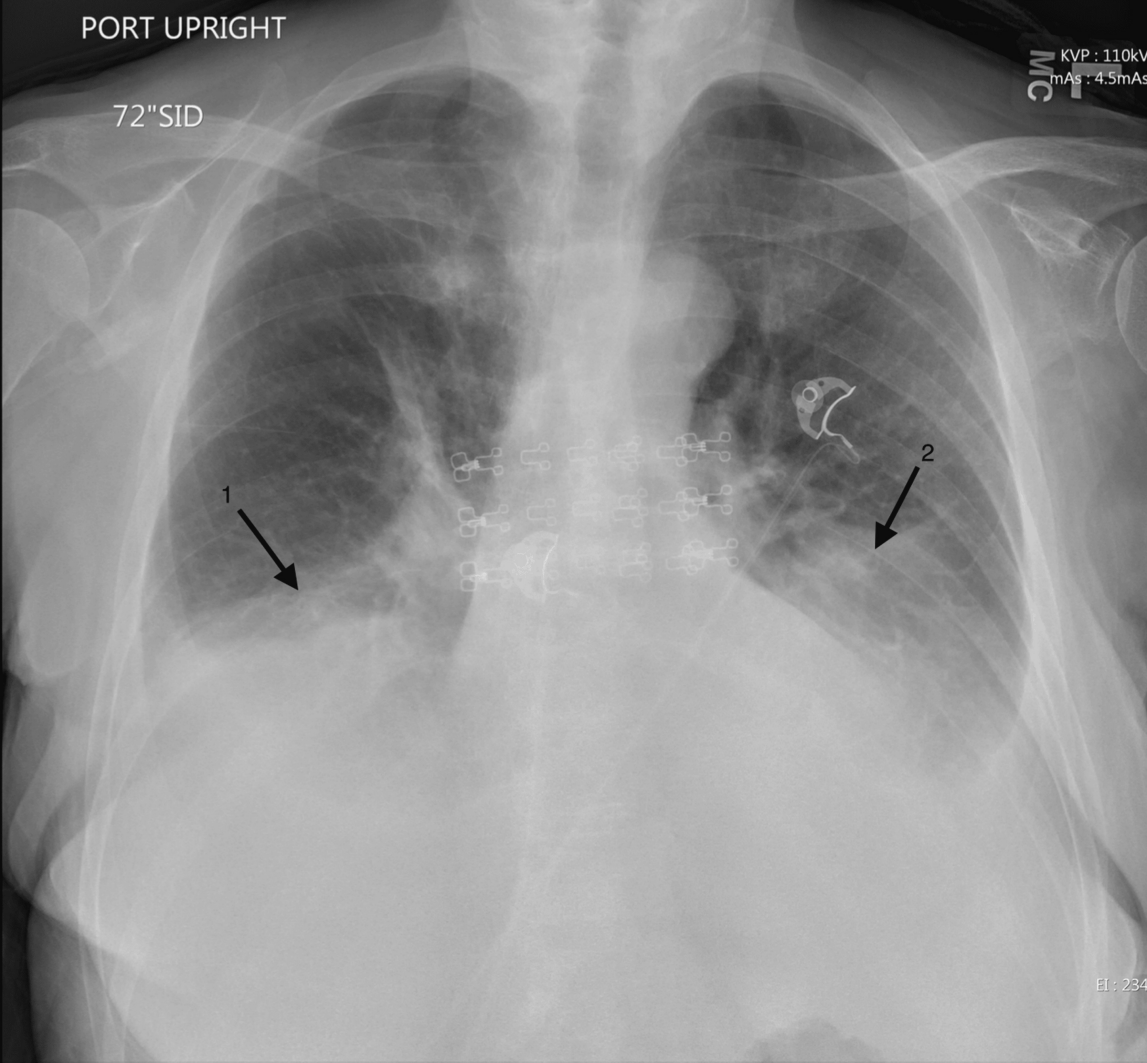
Chest radiograph on initial presentation identifying bilateral pleural effusions Arrows 1 and 2 display left and right pleural effusions, respectively. No pneumothorax was visualized

Imaging studies performed on the initial presentation included a chest radiograph and CT angiogram to rule out pulmonary embolism, congestive heart failure, pneumonia, and acute exacerbation of chronic obstructive pulmonary disease. Figure 2 demonstrates the CT angiogram of the chest, which identified moderate bilateral pleural effusions with atelectasis of bilateral lower lobes of the lungs. No pulmonary embolism was visualized; however, multiple small calcified granulomas were identified bilaterally. Initial chest radiograph, as seen in Figure 3, identified mild left basilar pleural-parenchymal disease with the absence of a pneumothorax.

**Figure 4:**
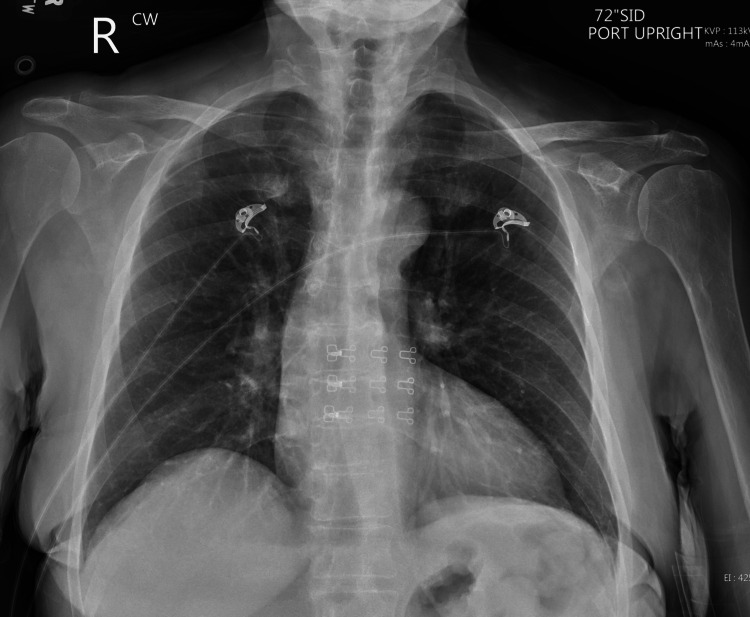
Chest radiograph following thoracentesis Resolved bilateral pleural effusions after diagnostic and therapeutic thoracentesis for the evaluation of pleural fluid

Following imaging, a thoracocentesis was performed. This was ordered to aid in identifying the underlying etiology of the pleural effusion via pleural fluid analysis. Distinguishing between the presence of an exudative or transudative effusion, performing cultures and gram staining/fungal staining for infection, aided in the process of identifying potential infectious, malignant, and inflammatory sources. Of note, 1450 mL of pleural fluid was collected via thoracocentesis from the right lower lobe and 1100 mL from the left lower lobe. Figure 4 displays the chest radiograph following thoracentesis with resolution of bilateral pleural effusions. 

Cultures of the collected fluid were obtained. No growth was observed at two days; however, there was a predominance of lymphocytes without organisms. No fungal element was seen on staining with calcofluor. This fluid collection, as shown in Tables 2-3, revealed an exudative effusion highly suggestive of a bilateral chylothorax secondary to elevated white blood cells with predominate lymphocytes and triglycerides. Given the patient’s history of CML, concurrent dasatinib therapy, and the absence of any other potential sources, the diagnosis of dasatinib-induced chylothorax was made. Dasatinib therapy was discontinued during admission and bosutinib therapy, an alternative BCR-ABL tyrosine kinase inhibitor, was initiated for the management of the patient’s CML. Post-hospitalization, the patient has been compliant with a non-fat diet. No recurrence of the chylothorax or acute events was observed at the six-month follow-up. 

**Table 2:** Pleural fluid analysis Pleural fluid analysis shows elevated triglycerides, cholesterol, predominant leukocytes, and red blood cells

Characteristic	Patient value	Reference range
Color and clarity	Pink milky fluid	Clear ultrafiltrate
White blood cells (WBCs/uL)	3,214	<1000
Red blood cells (RBCs/uL)	1,422	<100
pH	8.00	7.60-7.64
Triglycerides (mg/dL)	442	<110
Cholesterol (mg/dL)	66.0	<45
Lactate dehydrogenase, fluid (U/L)	127	<50% plasma
Glucose (mg/dL)	127	Equivalent to plasma
Albumin, fluid (g/L)	2.9	
Protein, fluid (g/L)	4.2	<2 g/dL

**Table 3:** Light's criteria Based on Light's criteria, the pleural effusions are likely exudative in etiology

Characteristic	Patient value	Exudative or transudative
Pleural/serum protein	0.617	Exudative
Pleural/serum lactate dehydrogenase	0.527	Exudative
Pleural fluid lactate dehydrogenase >2/3rds the upper limit of normal serum lactate dehydrogenase	127	Exudative

## Discussion

Pulmonary complications have been reported in approximately 35% of patients undergoing dasatinib therapy. Dasatinib, a second-generation BCR-ABL tyrosine kinase inhibitor, is often the preferred therapy for individuals undergoing treatment for Philadelphia-positive CML. The use of this specific tyrosine-kinase inhibitor has been associated with the development of chylothorax, a rare form of pleural effusion [7]. A chylothorax develops secondary to the accumulation of chyle or lymphatic fluid in the pleural cavity, originating from leakage of lymphatic channels and ducts. The predisposing factors include damage to the thoracic duct secondary to trauma, malignancies such as lymphoma or bronchogenic carcinoma, congenital lymphatic anomalies, or the use of certain medications [8].

The proposed pathologic process of dasatinib-induced chylothorax involves the creation of microscopic disruptions within the lymphatic channels, resulting in extravasation of chylous fluid [7]. Additionally, a regulatory tyrosine kinase protein of mesenchymal origin, platelet-derived growth factor beta (PDGF-β), is integral to the formation of the primitive cardiovascular system. PDGF-β is located adjacent to the granulocyte-macrophage colony-stimulating factor (GM-CSF) gene on chromosome 5 [9]. This integral positioning increases PDGF-β and GM-CSF expression, leading to uncontrolled proliferation of myeloid stem cell precursors and increased predisposition to the development of CML [9]. It is hypothesized that dasatinib, acting as a tyrosine kinase inhibitor, concomitantly inhibits PDGF-β expression, ultimately inducing increased lymphatic duct permeability [7]. Increased permeability enhances the release of chylous contents into the pleural space, subsequently leading to the development of a chylothorax. 

The most commonly reported symptom on initial presentation of a chylothorax, or pleural effusion in general, is dyspnea, as seen in our case [7]. Definitive diagnosis of a chylothorax, however, involves the application of Light’s criteria alongside additional pleural fluid analysis. Light’s criteria play a key role in distinguishing an exudative from a transudative pleural effusion. An exudative effusion is defined as a ratio of pleural protein to serum protein >0.5, the ratio of pleural lactate dehydrogenase (LDH) to serum LDH ratio >0.6, and pleural LDH greater than two-thirds of the normal upper limit. A chylothorax is further defined by a triglyceride level >110 mg/dL in pleural exudate.

An additional key feature, often characteristic of this form of pleural effusion, includes a predominance of lymphocytic cells on pleural fluid analysis [7]. Further diagnostic and confirmatory testing involves chest radiographs, mediastinal CT scans, and thoracentesis. Thoracentesis shows a collection of milky chylous fluid with an elevated triglyceride level, typically above 1.2 mmol/L. Pleural fluid analysis will display a total cholesterol level of <200 mg/dL, low LDH, and lymphocyte-predominant cells [10]. Our patient met the criteria for an exudative pleural effusion, alongside elevated triglycerides (Tables 2-3). The combination of these findings is consistent with the diagnosis of a chylothorax. Based on the patient’s history and lack of trauma, the most likely risk factor was the addition of dasatinib therapy for CML. 

Currently, the preferred management of a chylothorax is multimodal and ultimately depends on the underlying etiology. Generally, the treatment involves a combination of thoracentesis, dietary modification, pleurodesis, and potential thoracic duct ligation [8]. Our patient underwent a thoracentesis, both diagnostic and therapeutic. Dasatinib therapy was discontinued, while a different tyrosine kinase inhibitor, bosutinib, was initiated. Although bosutinib has a similar mechanism of action as dasatinib, serving as a BCR-ABL tyrosine kinase inhibitor, it has a unique feature that decreases the risk of development of a chylothorax in this patient population. As PDGFR serves as a molecular structure underlying the etiology of dasatinib-induced chylothorax, bosutinib fails to significantly antagonize this protein, thus decreasing the likelihood of the development of this adverse event [11].

During the follow-up, the patient adhered to a non-fat diet with no further recurrence of the chylothorax. New research has also suggested the addition of various hormones, including octreotide and somatostatin, to decrease gastric lymphatic flow and serve as a potential treatment for this form of pleural effusion [8]. These additional hormones would permit the slowing of chyle flow via the thoracic duct, minimizing and preventing the formation of a chylothorax.

## Conclusions

The development of chylothorax is a rare and poorly understood adverse effect of dasatinib therapy. Our patient presented with acute-onset dyspnea following initial therapy with dasatinib for the management of CML. Evaluation with imaging and labs at presentation identified bilateral pleural effusions, and was further confirmed to be a chylothorax with a diagnostic and therapeutic thoracentesis. Dasatinib therapy was discontinued and a non-fat diet was prescribed. The patient remained asymptomatic at the six-month follow-up appointment. Further investigations into the exact mechanism and pathologic process of dasatinib-induced chylothorax would advance our understanding and likely lead to the eventual prevention of this outcome. Raising awareness, through additional case reports, presentations, and studies, will provide further insights into potential risk factors and optimize the medical and procedural management of this rare pathology.
